# Dietary and Physical Activity Habits in Adolescent Girls with Polycystic Ovary Syndrome (PCOS)-HAstudy

**DOI:** 10.3390/jcm10163469

**Published:** 2021-08-05

**Authors:** Małgorzata Mizgier, Grażyna Jarząbek-Bielecka, Dorota Formanowicz, Elżbieta Jodłowska-Siewert, Kinga Mruczyk, Angelika Cisek-Woźniak, Witold Kędzia, Justyna Opydo-Szymaczek

**Affiliations:** 1 Department of Dietetics, Faculty of Physical Culture in Gorzów Wlkp., Poznan University of Physical Education, Estkowskiego 13, 66-400 Gorzów Wielkopolski, Poland; k.mruczyk@awf-gorzow.edu.pl (K.M.); a.cisek@awf-gorzow.edu.pl (A.C.-W.); 2 Department of Perinatology and Gynecology, Division of Developmental Gynecology and Sexology, Poznan University of Medical Sciences, 60-535 Poznan, Poland; grajarz@tlen.pl (G.J.-B.); witold.kedzia@poczta.fm (W.K.); 3 Chair and Department of Medical Chemistry and Laboratory Medicine, Poznan University of Medical Sciences, 60-806 Poznan, Poland; doforman@ump.edu.pl; 4 Department of Computer Science and Statistics, Poznan University of Medical Sciences, 60-806 Poznan, Poland; youleadmeastray@gmail.com; 5 Department of Pediatric Dentistry, Chair of Pediatric Dentistry, Poznan University of Medical Sciences, 60-812 Poznan, Poland; jopydo@ump.edu.pl

**Keywords:** diet, nutrition, physical activity, polycystic ovary syndrome, healthy lifestyle

## Abstract

The role of inappropriate lifestyle in the etiology of polycystic ovary syndrome (PCOS) and its metabolic and reproductive complications has attracted much attention in recent years; however, most studies involve adult patients. Thus, the study aimed to compare dietary patterns, physical activity, metabolic, anthropometric and inflammatory markers of 14–18-year-old girls with and without PCOS (n = 61 and n = 35, respectively) as well as to assess correlations between concentrations of metabolic and inflammatory markers and macronutrient intake and to identify the independent predictors of PCOS, related to diet and physical activity (PA). Compared to the control group, PCOS girls consumed significantly more total fat (*p* = 0.0005), including both saturated (SFA) (*p* = 0.03), monounsaturated (MUFA) (*p* = 0.0003) and polyunsaturated fatty acids (PUFA) (*p* = 0.01). A significantly higher percentage of PCOS patients consumed high and medium glycemic index (GI) foods (*p* = 0.03) and represented a low level of PA, both during school and in leisure time (41.67 vs. 6.06%; *p* = 0.0001 and 32.79 vs. 5.71%; *p* = 0.003, respectively). The PCOS group had also significantly higher waist circumference (WC), C-reactive protein (CRP) (*p* = 0.01), LDL cholesterol (*p* = 0.01), fasting insulin (*p* = 0.002) and HOMA-IR (*p* = 0.006) levels. There was an inverse correlation between fiber intake and fasting insulin, (*p* = 0.0002, r = −0.37), HOMA-IR (*p* = 0.0004, r = −0.35), WC (*p* = 0.029; r = −0.222) and a positive relationship between high and medium GI diet and insulin concentration (*p* = 0.003; r = 0.3). An increase of 10 g/day in total fat intake per day increases the probability of PCOS by 1.4 times. If the SFA or MUFA intakes increase by 10 g, the probability of PCOS increase 1.7-fold and 2.5-fold, respectively. The consumption of foods with a medium GI raises the probability of PCOS by more than 3 times, after adjusting for age. The odds ratio decreased for the moderate and high PA at school/work and in leisure time. Further research in girls with PCOS is needed to test whether low GI and dietary fatty acid reduction combined with increased PA is effective in the nonpharmacological treatment and prevention of PCOS complications. ClinicalTrial.gov Identifier: NCT04738409.

## 1. Introduction

Polycystic Ovary Syndrome (PCOS) is a common hormonal disorder affecting over 11% of teenage girls when using the Rotterdam diagnostic criteria [1,2,3]. A typical clinical feature of PCOS is hyperandrogenism, which is defined as the state of increased androgen production. It is manifested, among others, by amenorrhea or disorders of the menstrual cycle, moderate to severe acne and hirsutism [4,5]. PCOS is considered a female type of metabolic syndrome since it is often associated with obesity, insulin resistance, abnormal lipids profile and hypertension [2]. Components of metabolic syndrome and hormonal disturbances are associated with the increased risk of cardiovascular disease, infertility, endometrial cancer and may affect the composition of the oral and gut microbiome [3,6]. Due to chronic low-grade inflammation in the body, females with PCOS have increased levels of proinflammatory mediators and oxidative stress markers in the body fluids [4,5,7]. The causes of PCOS are not clearly understood; however, numerous studies have shown that environmental/lifestyle factors may modulate the secretion of hormones and affect the metabolic profile of the patients with endocrinopathy [8,9,10,11,12].

Behavioral and lifestyle modifications, such as diet and exercise, are primary treatments to promote fertility and reduce long-term health risks from gynecological diseases, including PCOS [13,14]. The International Evidence-based Guideline for the Assessment and Management of Polycystic Ovary Syndrome (PCOS) emphasizes the importance of diet and PA in the prevention of metabolic and reproductive complications related to PCOS and the role of weight control in overweight and obese PCOS patients. A 5% reduction in body weight is associated with an improvement in both metabolic and reproductive health markers, as well as with psychological benefits [15,16,17]. Patients with PCOS, with improper diet and low levels of PA, are probably more prone to weight gain than the population of healthy women [15,18,19]. As reminded by Eleftheriadou et al. and Teede et al., it is not clear whether the prevalence of obesity in the population may affect the prevalence of PCOS [19,20]. However, the longitudinal community based observational study shows that weight, BMI and 10-year weight gain were higher in PCOS [19].

The results of our previous observational studies indicate that the risk of excess body weight in teenage girls with PCOS is increased by both the occurrence of eating disorders and an improper diet, in particular, too low content of plant protein and consumption of carbohydrates with a high GI [21]. Moreover, our results revealed that carbohydrate intake, including the content of dietary fiber and protein intake, mainly of plant origin, are negatively correlated with markers of inflammation and oxidative stress, especially in overweight and obese girls with PCOS [22].

In the recommendations for adolescent female patients with PCOS for weight reduction, at least 60 min three times per week of moderate to vigorous PA, including muscle and bone-strengthening exercise, is recommended [15]. However, Stepto et al. highlights that an essential and urgent direction for further research involving girls with PCOS is to confirm the effect of a specific dimension (i.e., type and duration) of PA on improving clinical characteristics in these young women [15,23]. Moreover, the specific composition of the diet concerning the proportions of particular macronutrients that could be used in preventive and therapeutic interventions in adolescent patients with PCOS remains unclear, as the results of studies are often divergent [15,24]. In a pilot study by Stamets et al. involving adult women with PCOS, a one-month hypocaloric diet with low-protein and high-carbohydrate content resulted in a significant weight loss and significant improvement of reproductive and metabolic abnormalities [17]. The low-fat DASH diet has been proved to be effective in obese women with PCOS [25,26,27]. In contrast, the low glycemic load and glycemic index (GI) of the diet had positive effects on the cardiometabolic and reproductive profile of PCOS individuals [24]. In most studies, regardless of the proportion of macronutrients, a reduction in body weight was achieved in adult females with PCOS [28,29].

More research is needed in this area; mainly, most of the studies are conducted among adult PCOS patients [19,20,29,30,31,32]. In contrast, those conducted among young women do not give an unambiguous answer about the specific composition of the diet, which would be of therapeutic importance [33].

This study compares physical activity and the dietary pattern, including the quality of consumed carbohydrates, proteins (plant and animal), fats and cholesterol in young women with PCOS and healthy control girls. In addition, we aimed to check the correlations between the concentration of metabolic and inflammatory markers and macronutrient intakes. Moreover, we aimed to identify the independent predictors of PCOS, related to diet and PA.

We assumed that the results of our study would help plan the course of future dietary and PA interventions for girls with PCOS.

## 2. Materials and Methods

### 2.1. Study Design and Participants

This study, which was approved by the Bioethics Committee at the Poznań University of Medical Sciences (resolution no. 553/18, annex no. 161/20), is an observational cross-sectional, case-control study. This research activity was carried out as part of the project: “Ovarian Hyperandrogenism in Normal and Excessive Body Weight Adolescent Girls and Their Relation to Diet (HAstudy)”, registered at ClinicalTrails.gov (ClinicalTrials.gov Identifier: NCT04738409). The study was conducted in accordance with the Declaration of Helsinki. All patients and their parents signed a written consent for the girls’ participation in the study.

The study included 96 Caucasian patients aged 14 to 18 years. The study group (PCOS) consisted of 61 patients of the Gynecology and Perinatology Medical Clinic at the Gynecology and Obstetrics Hospital of Poznan University of Medical Sciences, who were diagnosed with PCOS according to the 2003 Rotterdam criteria, i.e., the presence of at least two of the following factors: clinical or biochemical hyperandrogenism, polycystic ovary image in an ultrasound examination, oligo-/amenorrhea [34]. The control group (CONTROL), which included 35 girls of the same age, was recruited during preventive examinations carried out in the Department of Developmental Gynecology and Sexology of Poznan University of Medical Sciences. The girls from the control group did not show any biochemical and clinical features of clinical hyperandrogenism (acne, hirsutism), the ultrasound examination was normal, adequate to the age and phase of the cycle.

We excluded patients with systemic diseases, thyroid dysfunction, diabetes mellitus, congenital adrenal hyperplasia, Cushing’s syndrome, hyperprolactinemia suggestive of pituitary adenoma, androgen-secreting tumors, taking medicines or hormonal contraceptives in the latest three months, taking vitamins or supplements, smoking. 

### 2.2. Nutritional Evaluation and Anthropometric Assessment

Anthropometric assessment of the patients was described in our previously published papers [21,22]. Evaluation of eating habits was carried out by a registered dietitian (M.M.) with the use of the three-day food records [35]. The participants recorded all food and beverages they ate and drank during three consecutive days. To assess the size of the portion consumed, girls used a photo album of the products and foods. Parents were asked to help their daughters record the diet. Data were analyzed using the Aliant software (Cambridge Diagnostics). The calculated daily food intake of each patient was compared with the daily nutritional and energy requirements according to the Human Nutrition Standards of the National Food and Nutrition Institute [36]. The procedure for nutritional evaluation has previously been described in detail in [21].

### 2.3. Biochemical Parameters

Blood tests and a gynecological examination with ultrasound assessment were carried out on the 3rd to 5th day of the cycle (apart from girls with secondary amenorrhea).

Blood levels of luteinizing hormone (LH), follicle stimulating hormone (FSH), sex hormone-binding globulin (SHBG), 17-β-estradiol, total testosterone, DHEA-S, insulin, glucose, triglycerides (TG), total cholesterol (TC), and (HDL-C), were measured in the morning in the fasting state. The analyses were performed in the central laboratory of the Gynecology and Obstetrics Hospital of Poznan University of Medical Sciences. Hormone levels and SHGB were determined by the electrochemiluminescence (ECLIA) immunoassay method (Elecsys) (Roche Diagnostics GmbH, Mannheim, Germany). Plasma TC, HDL-C, and TG levels were assessed by the enzymatic colorimetric method (Roche Diagnostics GmbH, Mannheim, Germany). Low-density lipoprotein cholesterol (LDL-C) concentration was calculated using Friedewald’s formula: LDL-C (mg/dL) = TC − (HDL-C + (TG/5). Plasma glucose was determined with the use of the enzymatic method with hexokinase.

Homeostasis model assessment for insulin resistance (HOMA-IR) was calculated using the following formula: HOMA-IR = fasting insulin (mU/mL) × fasting glucose (mmol/L)/22.5 [6,37].

Serum concentrations of the selected inflammatory markers (IL-6, IL-1, TNF-α, and CRP) were measured with the commercial enzyme-linked immunosorbent assay kits purchased from Shanghai Sunred Biological Technology Co. (Shanghai, China) using the TECAN-SUNRISE reader with the Magellan software in the Chair and Department of Medical Chemistry and Laboratory Medicine of Poznan University of Medical Sciences [22].

### 2.4. Physical Activity Assessment

The Beliefs and Eating Habits Questionnaire (KomPAN), developed and validated by the Commission of Behavioral Determinants of Nutrition from the Polish Academy of Sciences (Warsaw, Poland) was used in the study to collect information on PA. The reproducibility of the interviewer-administered questionnaire (KomPAN) in Polish adolescents and adults was evaluated by Kowalkowska et al. [38].

The questionnaire contained two questions concerning activity: PA at work/school and PA at leisure time.

Each of the questions concerning PA had three response categories: low, moderate, high PA. The description of the scale was presented separately for the PA in leisure and work/school time. For leisure time, ‘low’ means ‘sedentary lifestyle, watching TV, reading the press and books, light housework, taking a walk for 1–2 h a week’; ‘moderate’ means’walks, cycling, gymnastics, gardening or other light PA performed for 2–3 h a week‘; and ‘high’ means ‘cycling, running, working on a plot or garden and other sports activities requiring physical effort, taking up more than 3 h a week’. ‘Low’ activity at work/school time was described as ‘over 70% of the time in a sitting position’, ‘moderate’ as ‘approximately 50% the of the time in a sitting position and about 50% of the time moving’, and ‘high’ as ‘about 70% of the time in motion or doing physical work associated with much effort’ [38].

### 2.5. Statistical Analysis

Statistical analysis was conducted using PQStat v.1.8.0 software. Depending on the type of data, descriptive statistics were presented as mean and standard deviation (sd), median and quartiles or numbers and percentages. The normal distribution of quantitative data was verified using the Shapiro–Wilk test. When comparing two groups, the unpaired t-test was used for normally distributed data, and for ordinal data or data that was not normally distributed, the Mann–Whitney test was applied. For analyzing correlations, the Pearson correlation coefficient was calculated for normally distributed variables and the Spearman’s rank correlation coefficient for ordinal and not normally distributed data. Univariate and multivariate logistic regression was used to determine the predictors of developing PCOS. Univariate models were built for all variables that were significantly different between the PCOS group and healthy controls. Subsequently, a multivariate model was built comprising all variables that were not strongly interconnected between one another to avoid multicollinearity. In this model age, BMI, CRP, LDL-C, total fat intake, glycemic index, work/school physical activity and leisure time physical activity were independent variables, and this model was used to calculate adjusted odds ratio (AOR) for macronutrients intake. Moreover, two other multivariate logistic regression models were created, with age and age and BMI as independent variables, respectively.

A *p*-value of 0.05 was considered statistically significant during analysis.

## 3. Results

The characteristics of anthropometric, biochemical and metabolic parameters of the girls participating in the study are presented in Table 1. Study groups: PCOS and control groups did not differ significantly in age, height and body weight. Waist circumference was significantly higher in girls with PCOS (65.5 vs. 76 cm) (Table 1).

PCOS patients showed statistically significant differences compared to the control group regarding serum concentration of metabolic parameters such as LDL-C (*p* = 0.01) and fasting insulin (*p* = 0.002). In the study group the insulin resistance index HOMA-IR and the CRP concentration were significantly higher if compared to the controls (*p* = 0.006 and *p* = 0.01, respectively). The differences between the groups including other tested metabolic and inflammatory parameters were not statistically significant (Table 1).

Regarding diet, the PCOS group differed significantly from the control group in fat consumption (*p* < 0.001), including both saturated fatty acids-SFA (*p* = 0.03), monounsaturated fatty acids-MUFA (*p* < 0.001) and polyunsaturated fatty acids PUFA (*p* = 0.01). Cholesterol consumption seemed to be also higher in the PCOS group, but it was not a statistically significant difference (223.5 vs. 199.56 g) (*p* = 0.749). There were no significant differences in energy intake, protein intake or carbohydrates intake between groups. On the other hand, in the study group, a significantly greater percentage of patients consumed products with a high and medium glycemic index (GI) (*p* = 0.03), and in the control group-products with a low GI (*p* = 0.03), which are usually characteristic of food products with higher fiber content and complex carbohydrates. The fiber content in the diet of PCOS group was 3 g lower (15.3 g vs. 18.08 g), while the consumption of added sugars was slightly higher in this group (31.7 g vs. 30.77 g) (Table 2).

Significantly more patients from the PCOS group declared low PA than the control group both during school/work (work/school PA) and leisure time (PA) (41.67 vs. 6.06%; *p* < 0.001 and 32.79% vs. 5.71%; *p* = 0.003, respectively). There were more girls with high PA in the group of healthy subjects, but the difference was not statistically significant (Table 2).

Moreover, a significant correlation was found between the intake of specific macronutrients and concentrations of metabolic parameters such as TG, HDL-C, fasting glucose and insulin, and HOMA-IR index (Table 3). Plant protein intake negatively correlated with fasting glucose (*p* = 0.05, r = −0.21) and insulin levels (*p* = 0.006, r = −0.28) and HOMA-IR index (*p* = 0.004, r = −0.29). An inverse correlation was also observed between dietary carbohydrate intake and TG concentration (*p* = 0.02, r = −0.24). Dietary fiber intake showed an inverse correlation with fasting insulin concentration (*p* < 0.001, r = −0.37), HOMA-IR (*p* < 0.001, r = −0.35) and waist circumference (*p* = 0.029; r = −0.222) (Table 3).

As presented in Table 4, total fat intake, and high leisure physical activity (PA) were found to be associated with PCOS, independently of each other and age, BMI, CRP, LDL-C, GI and work/school PA. The odds ratio of PCOS increases 1.4-fold (1.53-fold after adjusting for age and BMI, and 1.18-fold after adjusting for age, BMI, CRP, LDL-C, GI, work/school PA and leisure PA) when total fat intake increases by 10 g. Furthermore, an increase of 10 g/day of SFA increases the probability of PCOS by almost 1.7-fold (more than 2-fold after adjusting for age and BMI, and more than 2.3-fold after adjusting for age, BMI, CRP, LDL-C, GI, work/school PA, and leisure PA). The odds ratio increases 2.5-fold when MUFA intake increases by 10 g/day (2.7-fold after adjusting for age and BMI, and 3.3-fold after adjusting for age, BMI, CRP, LDL-C, GI, work/school PA, and leisure PA).

While low-GI does not significantly increase the probability of PCOS, the consumption of medium-GI products increases the probability of PCOS by more than 3-fold when adjusted for the girls’ age (Table 4).

Finally, moderate to high PA in both leisure time PA and work/school PA significantly reduces the probability of PCOS (OR = 0.15, *p* = 0.02; OR = 0.09, *p* = 0.004, respectively, and OR = 0.12, *p* = 0.006; OR = 0.04, *p* = 0.0005 respectively), independent of age and BMI. Moreover, high leisure PA significantly reduces the probability of PCOS, independently of age, BMI, CRP, LDL-C, total fat intake, GI, work/school PA (Table 4).

## 4. Discussion

The girls with PCOS differed significantly from healthy age-mates in the intake of specific macronutrients and PA in free time and at school/work. Taking into account the diet, the girls from the PCOS group showed significantly higher consumption of total fats, including both SFA, MUFA and PUFA fatty acids, as compared to the control group. In multivariate analysis, total fat intake and high leisure PA were found to be associated with PCOS, independently of each other and age, BMI, CRP, LDL-C, GI and work/school PA. Our results also indicate that an increase in total fat intake, SFA and MUFA 10 g per day increase the probability of PCOS by 1.4-fold, 1.7-fold (2.3 after adjusting for age, BMI, CRP, LDL-C, GI, work/school PA, and leisure PA), and 2.5-fold (3.3-fold after adjusting for age, BMI, CRP, LDL-C, GI, work/school PA, and leisure PA), respectively.

As shown by the results of other studies, a diet rich in fat, particularly saturated fat, increases the risk of insulin resistance and intensifies the complications associated with it [39,40,41]. Moreover, cholesterol in the diet of slim girls with PCOS shows a positive correlation with the concentration of the inflammatory marker–CRP [22], whereas a diet with a low-fat content, mainly SFA, has a favorable effect on the metabolic profile [25,31], including the lipid profile [27]. At the same time, animal studies have shown that PUFA fatty acids reduce the risk of insulin resistance [42,43,44]. Similar results were obtained in adult patients with non-alcoholic fatty liver disease [45]. On the contrary, the study by Kasim-Karakas et al., in which patients with PCOS were subjected to three-month dietary intervention, proved that increasing the proportion of PUFA in the diet had a negative effect on the concentration of fasting glucose and the concentration of glucose in the oral glucose tolerance test (OGTT) [46]. An increase in fasting glucose in response to an increase in PUFA consumption was also observed in a study by Madigan et al., conducted in patients with type 2 diabetes [47]. It has been proven that a high-fat diet, regardless of the type of fat (PUFA, MUFA or SFA), can also be a factor in the development of gestational diabetes [48]. It is worth noting that the common feature that plays a role in the pathomechanism of gestational diabetes, type 2 diabetes and PCOS is the presence of insulin resistance with accompanying insulin deficiency [21,48,49]. Interventional studies, based on patients with type 2 diabetes, indicate that the effect of PUFA on glucose concentration may be more beneficial than that of SFA, but replacing saturated and polyunsaturated fatty acids with monounsaturated oleic acid, typical for the Mediterranean diet, which is abundant in olive oil, has a much better effect [50]. In the study by Ryan et al., the change in the fatty acid profile from polyunsaturated to monounsaturated decreased both fasting glucose and insulin and, at the same time, insulin resistance in the subjects [50]. The Mediterranean diet was found to be significantly associated with lower insulin concentrations, lower HOMA-IR index values, and increased tissue insulin sensitivity [51], lower fasting glucose and insulin levels in obese patients [52], as well as lower BMI and waist circumference [51]. However, when formulating conclusions from interventional studies in patients following the Mediterranean diet, it should be remembered that apart from olive oil, other components of the Mediterranean diet, such as fiber or a large load of antioxidants present in vegetables and fruits or fiber, may also contribute to the improvement of metabolic parameters and inflammatory markers [53,54].

In our study, both groups differed significantly in terms of the GI of the diet. In the PCOS group, a significantly higher percentage of patients consumed products with a high and medium GI (*p* = 0.03), and in the control group products with a low GI (*p* = 0.03). Our study also showed that the consumption of foods with a medium GI increases the probability of PCOS by more than 3-fold (after taking into account the age of the subjects). It is worth mentioning that low GI is usually characterized by food products with higher fiber content and complex carbohydrates [55]. We also noticed a not statistically significant higher consumption of added sugars and lower dietary fiber content in the PCOS group. The median consumption in this group was only 15 g/day. Nevertheless, the supply of fiber in both groups was below the lower limit of the normal consumption, which is 20 g/day [56]. We also observed that fiber intake was negatively correlated with metabolic markers, such as glucose, insulin and waist circumference. Moreover, as shown by our study, girls with PCOS, despite their young age, differed significantly from the control group in terms of metabolic indicators, such as waist circumference (*p* < 0.000001), serum LDL-C (*p* = 0.01), fasting insulin (*p* = 0.002) and the HOMA-IR index (*p* = 0.006), which assesses insulin resistance based on the relationship between insulin and blood glucose levels. There was also a statistically significant difference in the CRP in the two groups (*p* = 0.01).

Studies showed that insufficient fiber intake in PCOS is important for the incidence of insulin resistance, cardiovascular disease and reproductive functions [57,58], and that consumption of saturated fat and cholesterol adversely affects the metabolic profile typical for PCOS [59]. Khayyatzadeh et al. found that a low-fiber diet increased inflammation in the body and serum CRP levels [60]. On the other hand, higher fiber consumption is associated with reduced inflammation in adult women [61], including lower serum CRP levels in patients with PCOS [62]. Moreover, it has been proved that the consumption of high GI products has been associated with an increase in inflammation in patients with hyperandrogenism and insulin resistance [63]. The research conducted in the present study indicates that high dietary GI is positively correlated with fasting insulin levels and the HOMA-IR index. Our previous research also showed that the probability of excess body weight was several times higher when consuming products with medium and high GI. We also found that a low GI diet was four times less common in overweight and/or obese PCOS girls than PCOS patients with normal body weight [21]. Other researchers also reported the beneficial effects of a low GI diet and its positive impact on weight loss, glucose concentration and hormonal profile in patients with PCOS [64,65,66,67,68,69,70].

The beneficial modulation of body weight in girls with PCOS may be due not only to the quality of dietary carbohydrates, including the GI value but also plant protein content in the diet. Previous studies have shown that an increase in plant protein intake by 10 g/day reduced the likelihood of overweight and obesity in girls with PCOS and excess body weight [21]. Moreover, plant protein intake was inversely correlated with IL-6, TNF-α in girls with PCOS, and excess body weight. A negative correlation was also observed between the total protein intake in PCOS patients and between overweight and obesity and the concentration of IL-6, TNF-α [22]. Nevertheless, in the present study, girls with PCOS did not differ in protein consumption, both plant and animal, compared to the control group. However, we observed that plant protein intake was inversely correlated with concentrations of metabolic markers: fasting glucose and insulin and HOMA-IR index.

### Physical Activity

According to the latest recommendations, PA, reduction and maintenance of proper body weight are essential elements of the therapy of metabolic changes associated with PCOS [15,71]. In a pilot study, intensive PA affected metabolic hormonal health in women with PCOS [72]. PA improves insulin sensitivity and the functioning of mitochondria [73]. In our study, both during work/school and in free time, significantly more patients from the PCOS group declared low PA than girls from the control group (41.67 vs. 6.06%, respectively; *p* = 0. 0001 and 32.79% vs. 5.71%; *p* = 0.003). Moreover, more girls with high PA were observed in the control group. Similarly to the study of Eleftheriadou et al., healthy girls were more likely to engage in PA than their peers with PCOS. The frequency and intensity of exercise in girls with PCOS were lower compared to healthy adolescent girls. Furthermore, adolescents with PCOS were less likely to be aware of the positive effects of exercise on their health [20]. The results of our study indicate that engaging in moderate to vigorous PA during both leisure time (leisure PA) and school/work PA significantly reduces the probability of PCOS (OR = 0.15, *p* = 0.02; OR = 0.09, *p* = 0.004, respectively and OR = 0.12, *p* = 0.006; OR = 0.04, *p* = 0.0005). After adjusting for age and BMI, this odds ratio decreases significantly, for both high and moderate PA. Moreover, high PA in leisure time significantly reduces the probability of PCOS, independently of age, BMI, CRP, LDL-C, total fat intake, GI and work/school PA.

The study has some limitations which should be considered in the interpretation of the results. Firstly, we used a questionnaire to characterize PA, so it was a subjective assessment. Future studies would be necessary to check, using objective measurements, such as an accelerometer, whether and what PA improves the metabolic and hormonal profile. Specific patterns should be created regarding the level of PA, the length of a single session and the frequency of exercises.

Secondly, the three-day food records method may be associated with incorrect portion size estimation or other bias related to subjective consumption assessment [74,75]. We partially addressed this problem by asking parents to help their daughters record the diet, which reduced the risk of overestimating or underestimating the portion size.

Another limitation of our research is their observational, not interventional, nature, so we cannot draw cause-and-effect conclusions regarding the potential impact of a diet high in fat and high and medium GI products and low PA on PCOS development.

Finally, in this current study, we aimed to identify the independent predictors of PCOS, related to diet and PA but hyperandrogenism may also predispose to differences in the quality and the amount of nutrients intake, which has been studied in the research of Kanaya et al. [76].

## 5. Conclusions

The research results indicate that in lifestyle of PCOS patients and the control group, age-mates differ significantly. Girls with PCOS had a significantly higher intake of fat, including SFA, MUFA, and PUFA, as compared to the control group. In addition, a substantially higher percentage of patients with PCOS consumed products with a high and medium GI and consumed lower amounts of fiber. Moreover, girls with PCOS showed low PA both at school and in their free time. Our results also indicate that an increase of 10 g/day in total fat intake increases the probability of PCOS by 1.4-fold, of SFA by 2-fold (2. 5-fold after adjusting for age and BMI) and an increase in MUFA intake by 10 g/day increases the probability of PCOS by 2.5-fold. The odds ratio increased more than 3 times for the consumption of products with a medium GI, after adjusting for age. In contrast, the probability of PCOS significantly decreases when both high and moderate PA is undertaken.

At the same time, girls with PCOS had significantly higher values of WC, LDL-C, fasting insulin, HOMA-IR index and the concentration of the inflammatory marker-CRP.

Our results may guide the therapeutic goals of gynecologists and other health professionals, who should encourage and help adolescent girls to change their lifestyle, including diet and PA level.

Further research in adolescent girls with PCOS is warranted to test whether non-pharmacological intervention with low GI and dietary fatty acids reduction in the daily intake and simultaneously increased PA is effective in the treatment and prevention of PCOS complications.

## Figures and Tables

**Table 1:** Anthropometric, metabolic, inflammatory markers and PA comparison between PCOS group (PCOS) and control group (CONTROL).

Variables	PCOS n = 61	CONTROL n = 35	*p*-Value
Age (years)			0.125
median (25–75%)	16 (15–17)	15 (15–17)	
Body height (m)			0.876
mean(sd)	166.2(6.09)	166.4(6.17)	
Body weight (kg)			0.159
median (25–75%)	62.3 (52.8–77.5)	56.9 (53.25–62.25)	
BMI categories			**0.001**
Underweight	2 (3.28%)	0	
Healthy weight	37 (60.66%)	34 (97.14%)	
Overweight/Obesity	22 (36.07%)	1 (2.86%)	
IL-1 (pg/mL)			0.116
median (25–75%)	24.7 (18.93–32.35)	22.75 (15.09–29.87)	
IL-6 (ng/L)			0.189
median (25–75%)	28.15 (22.72–36.89)	24.33 (18.19–43.15)	
TNF-α (ng/L)			0.313
median (25–75%)	74.54 (56.37–107.8)	85.16 (64.8–98.2)	
CRP (mg/L)			**0.010**
median (25–75%)	0.71 (0.35–1.3)	0.41 (0.28–0.58)	
TC (mg/dL)			0.096
median (25–75%)	158 (135.2–174.4)	145.3 (125.6–165.4)	
LDL-C (mg/dL)			**0.013**
median (25–75%)	80.3 (64.3–98.2)	65.4 (57.6–81.15)	
HDL-C (mg/dL)	55.42(10.4)	58.27(14.07)	0.300
mean(sd)			
TG (mg/dl)			0.228
median (25–75%)	84.5 (66.7–117.4)	75.6 (58.05–106.45)	
Fasting glucose (mmol/L)			0.942
median (25–75%)	88.1 (84.48–92.5)	90.4 (81.75–93.75)	
Fasting insulin (mU/mL)			**0.002**
median (25–75%)	14.36 (9.35–19.11)	9.74 (7.73–13.06)	
HOMA-IR			**0.006**
median (25–75%)	2.94 (1.87–4.36)	2.24 (1.64–2.8)	
WC (cm)			**<0.001**
median (25–75%)	76 (68–87)	65.5 (64.25–67)	

Abbreviations: PCOS, polycystic ovary syndrome; IL-1-interleukin-1; IL-6-interleukin-6; TNF-α-Tumor Necrosis Factor α; CRP-C-reactive protein; TC-total cholesterol; LDL-C—low-density lipoprotein cholesterol; HDL-C—high-density lipoprotein cholesterol; TG-triglycerides; HOMA-IR-Homeostatic Model Assessment of Insulin Resistance; WC—Waist Circumference. All the *p*-values marked in bold were statistically significant.

**Table 2:** Diet composition and physical activity comparison between PCOS group (PCOS) and control group (CONTROL).

Variables	PCOS n = 61	CONTROL n = 35	*p*-Value
Total energy (kcal)			0.081
median (25–75%)	1663.5 (1444.7–1788.4)	1474.01 (1189.44–1746.39)	
Total protein (g)			0.825
median (25–75%)	67.3 (53.6–84.3)	68.81 (61.47–78.69)	
Total fat (g)			**<0.001**
mean(sd)	62.93(24.68)	47.42(16.97)	
Total carbohydrate (g)			0.709
median (25–75%)	213.6 (184.3–231)	199.91 (165.61–240.1)	
Fiber (g)			0.069
median (25–75%)	15.3 (11.1–20.2)	18.08 (14.53–22.2)	
Added sugars (g)			0.703
median (25–75%)	31.7 (21.6–42.5)	30.77 (19.79–41.55)	
Animal protein (g)			0.223
median (25–75%)	37.8 (31.2–50)	44.39 (37.89–51.6)	
Plant protein(g)			0.825
median (25–75%)	21.8 (17.2–25.1)	21.32 (18.94–26.02)	
SFA og, (g)			**0.034**
mean(sd)	24.57(10.13)	20.26(8.15)	
MUFA (g)			**<0.001**
mean(sd)	22.2(9.73)	16.32(5.78)	
PUFA (g)			**0.014**
median (25–75%)	7.5 (5.2–10.6)	5.52 (4.71–6.92)	
Cholesterol (mg)			0.749
median (25–75%)	223.5 (167.7–301.2)	199.56 (155.52–345.05)	
GI			**0.030**
low	14 (22.95%)	15 (42.86%)	
medium	45 (73.77%)	20 (57.14%)	
high	2 (3.28%)	0	
Work/school PA			**<0.001**
low	25 (41.67%)	2 (6.06%)	
moderate	29 (48.33%)	20 (60.61%)	
high	6 (10%)	11 (33.33%)	
Leisure PA			**0.003**
low	20 (32.79%)	2 (5.71%)	
moderate	27 (44.26%)	18 (51.43%)	
high	14 (22.95%)	15 (42.86%)	

Abbreviations: PCOS, polycystic ovary syndrome; SFA, saturated fatty acid; MUFA, monounsaturated fatty acids; PUFA, polyunsaturated fatty acids; GI, glycemic index; PA, physical activity; All the *p*-values marked in bold were statistically significant.

**Table 3:** Spearman and Pearson’s correlations among energy, macronutrients intake and inflammatory and metabolic markers in adolescent girls.

Variables	Total Protein (g)	Total Fat (g)	Total Carbohydrate (g)	Fiber (g)	Plant Protein (g)	SFA (g)	MUFA (g)	PUFA (g)	Cholesterol (mg)	GI
IL-1 *p* value	0.580	0.371	0.690	0.465	0.919	0.503	0.225	0.436	0.372	0.560
r	−0.057	0.092	−0.041	−0.076	0.011	0.069	0.125	0.080	0.092	0.060
IL-6 *p* value	0.907	0.156	0.709	0.806	0.356	0.324	0.080	0.125	0.104	0.679
r	0.012	0.146	0.039	0.025	0.095	0.102	0.180	0.158	0.167	0.043
TNF-α *p* value	0.788	0.915	0.532	0.934	0.984	0.814	0.857	0.698	0.545	0.927
r	−0.028	0.011	−0.065	−0.009	−0.002	0.024	0.019	0.040	0.062	0.009
CRP *p* value	0.238	0.978	0.424	0.140	0.454	0.825	0.622	0.996	0.542	0.079
r	−0.121	−0.003	−0.082	−0.152	−0.077	−0.023	0.051	−0.001	0.063	0.180
TC *p* value	0.680	0.811	0.310	0.308	0.404	0.665	0.793	0.845	0.544	0.055
r	−0.043	0.025	−0.105	−0.105	−0.086	0.045 *	0.027	0.020	0.063	0.197
LDL-C *p* value	0.816	0.352	0.826	0.309	0.833	0.847	0.322	0.277	0.535	0.054
r	−0.024	0.096	−0.023	−0.105	−0.022	0.020	0.102	0.112	0.064	0.197
HDL-C *p* value	0.772	0.919	0.775	0.972	0.892	0.563	0.737	0.627	0.510	0.410
r	0.030	0.011	−0.030	0.004	−0.014	0.060 *	−0.035	−0.050	0.068	0.085
TG *p* value	0.113	0.483	**0.017**	0.248	0.073	0.477	0.447	0.457	0.517	0.390
r	−0.163	−0.072	**−0.243**	−0.119	−0.184	−0.073	−0.079	−0.077	−0.067	0.089
Fasting glucose *p* value	0.687	0.167	0.397	0.146	**0.045**	0.192	0.329	0.590	0.091	0.443
r	0.042	0.143	−0.088	−0.150	**−0.207**	0.135	0.101	0.056	0.174	0.080
Fasting insulin *p* value	0.218	0.721	0.122	**<0.001**	**0.006**	0.866	0.654	0.959	0.864	**0.003**
r	−0.127	0.037	−0.159	**−0.366**	**−0.276**	−0.017	0.046	0.005	−0.018	**0.302**
HOMA-IR *p* value	0.368	0.994	0.074	**<0.001**	**0.004**	0.774	0.958	0.766	0.709	**0.017**
r	−0.093	−0.001	−0.183	**−0.353**	**−0.290**	−0.030	−0.005	−0.031	0.039	**0.243**
WC *p* value	0.686	0.236	0.616	**0.029**	0.224	0.964	0.101	0.182	0.535	0.334
r	−0.042	0.122	−0.052	**−0.222**	−0.125	−0.005	0.169	0.137	−0.064	0.100

Abbreviations: SFA, saturated fatty acid; MUFA, monounsaturated fatty acids; PUFA, polyunsaturated fatty acids; GI, glycemic index; IL-1—Interleukin-1; IL-6—Interleukin-6; TNF-α—Tumor Necrosis Factor; CRP—C-reactive protein; TC—total cholesterol, LDL-C—low-density lipoprotein cholesterol, HDL-C—high-density lipoprotein cholesterol, TG—triglycerides, HOMA-IR—Homeostatic Model Assessment of Insulin Resistance; WC—Waist Circumference. The r coefficients are either Spearman’s correlation coefficients (not marked) or Pearson’s correlation coefficients (r marked with an asterisk). All the *p*-value marked in bold were statistically significant.

**Table 4:** The odds ratio of macronutrients intake, GI and PA and the risk of PCOS.

	Odds Ratio of PCOS							
	OR (95% CI)	*p-*Value	AOR (95% CI)*	*p-*Value *	AOR (95% CI) **	*p-*Value **	AOR (95% CI) ***	*p-*Value ***
Total protein (per 1 g)	1.006 (0.987–1.025)	0.536	1.003 (0.983–1.023)	0.787	1.004 (0.983–1.025)	0.712	0.977 (0.934–1.022)	0.311
Total protein (per 10 g)	1.061 (0.880–1.280)	0.536	1.028 (0.841–1.258)	0.787	1.040 (0.844–1.283)	0.712	0.792 (0.506–1.243)	0.311
Total fat (per 1 g)	1.036 (1.012–1.060)	**0.003**	1.034 (1.010–1.059)	**0.006**	1.043 (1.014–1.074)	**0.004**	1.062 (1.020–1.106) ****	0.004 ****
Total fat (per 10 g)	1.421 (1.129–1.789)	**0.003**	1.398 (1.102–1.773)	**0.006**	1.530 (1.145–2.046)	**0.004**	1.183 (1.217–2.745) ****	0.004 ****
Total carbohydrates (per 1 g)	1.002 (0.996–1.008)	0.565	1.000 (0.993–1.007)	0.989	1.000 (0.993–1.008)	0.902	0.995 (0.983–1.007)	0.400
Total carbohydrates (per 10 g)	1.019 (0.957–1.085)	0.565	1.000 (0.933–1.071)	0.989	1.005 (0.932–1.084)	0.902	0.950 (0.843–1.071)	0.400
Fiber (per 1 g)	0.992 (0.951–1.035)	0.713	0.972 (0.928–1.019)	0.238	0.980 (0.933–1.030)	0.424	0.974 (0.891–1.066)	0.572
Fiber (per 10 g)	0.923 (0.602–1.415)	0.713	0.755 (0.473–1.204)	0.238	0.818 (0.500–1.339)	0.424	0.772 (0.314–1.895)	0.572
Plant protein (per 1 g)	1.008 (0.865–1.052)	0.727	0.994 (0.945–1.043)	0.801	1.005 (0.954–1.060)	0.841	0.973 (0.902–1.051)	0.489
Plant protein (per 10 g)	1.080 (0.702–1.661)	0.727	0.940 (0.579–1.524)	0.801	1.055 (0.624–1.785)	0.841	0.764 (0.356–1.637)	0.489
SFA (per 1 g)	1.052 (1.003–1.104)	**0.039**	1.053 (1.000–1.108)	**0.049**	1.075 (1.011–1.143)	**0.022**	1.088 (1.001–1.182) *****	0.048 *****
SFA (per 10 g)	1.666 (1.027–2.700)	**0.039**	1.672 (1.002–2.789)	**0.049**	2.053 (1.112–3.792)	**0.022**	2.319 (1.009–5.329) *****	0.048 *****
MUFA (per 1 g)	1.096 (1.031–1.165)	**0.003**	1.088 (1.023–1.158)	**0.007**	1.105 (1.026–1.192)	**0.009**	1.127 (1.012–1.254) *****	0.029 *****
MUFA (per 10 g)	2.506 (1.364–4.604)	**0.003**	2.331 (1.257–4.322)	**0.007**	2.725 (1.287–5.768)	**0.009**	3.296 (1.126–9.648) *****	0.029 *****
PUFA (per 1 g)	1.114 (0.996–1.246)	0.060	1.090 (0.968–1.228)	0.155	1.091 (0.968–1.230)	0.155	1.136 (0.976–1.321) *****	0.099 *****
PUFA (per 10 g)	2.938 (0.957–9.019)	0.060	2.375 (0.722–7.813)	0.155	2.390 (0.719–7.944)	0.155	3.575 (0.788–16.223) *****	0.099 *****
Cholesterol (per 1 mg)	0.999 (0.997–1.002)	0.661	1.000 (0.997–1.002)	0.793	1.000 (0.997–1.003)	0.946	0.997 (0.991–1.003)	0.315
Cholesterol (per 10 mg)	0.994 (0.970–1.020)	0.661	0.996 (0.970–1.023)	0.793	1.000 (0.972–1.027)	0.946	0.971 (0.916–1.029)	0.315
GI (low)	Reference		Reference		Reference		Reference	
GI (moderate)	2.411 (0.981–5.923)	0.055	3.035 (1.132–8.138)	**0.027**	2.437 (0.867–6.850)	0.091	3.68 (0.919–14.754) ****	0.066 ****
GI (high)	Not enough data		Not enough data		Not enough data		Not enough data	
Work/school PA (low)	Reference		Reference		Reference		Reference	
Work/school PA (moderate)	0.116 (0.025–0.546)	**0.006**	0.127 (0.026–0.618)	**0.011**	0.174 (0.035–0.859)	**0.032**	0.655 (0.085–5.031) ****	0.685 ****
Work/school PA (high)	0.044 (0.008–0.251)	**0.0005**	0.051 (0.008–0.310)	**0.001**	0.062 (0.010–0.385)	**0.003**	0.611 (0.060–6.238) ****	0.677 *****
Leisure PA (low)	Reference		Reference		Reference			
Leisure PA (moderate)	0.150 (0.031–0.722)	**0.018**	0.143 (0.027–0.748)	**0.021**	0.172 (0.032–0.916)	**0.039**	0.162 (0.017–1.563) ****	0.116 ****
Leisure PA (high)	0.093 (0.018–0.474)	**0.004**	0.058 (0.010–0.340)	**0.002**	0.080 (0.013–0.478)	**0.006**	0.030 (0.002–0.438) ****	0.010 ****

Abbreviations: SFA, saturated fatty acid; MUFA, monounsaturated fatty acids; PUFA, polyunsaturated fatty acids; GI, glycemic index; PA, physical activity; * The odds ratio adjusted for age; ** The odds ratio adjusted for age and BMI; *** The odds ratio adjusted for age, BMI, CRP, LDL-C, total fat intake, glycemic index, work/school physical activity and leisure time physical activity; **** Variables included in the model as independent variables; ***** Due to a very strong correlation between SFA, MUFA, PUFA and total fat intake, AOR for SFA, MUFA and PUFA were calculated in the model excluding total fat intake as an independent variable. All the *p*-values marked in bold were statistically significant.

## Data Availability

The datasets generated during and/or analyzed during the current study are available from the corresponding author on reasonable request.

## References

[1] Naz M.S.G., Tehrani F.R., Majd H.A., Ahmadi F., Ozgoli G., Fakari F.R., Ghasemi V. (2019). The prevalence of polycystic ovary syndrome in adolescents: A systematic review and meta-analysis. Int. J. Reprod. Biomed..

[2] Sam S., Dunaif A. (2003). Polycystic ovary syndrome: Syndrome XX?. Trends Endocrinol. Metab..

[3] Zhao X., Jiang Y., Xi H., Chen L., Feng X. (2020). Exploration of the Relationship Between Gut Microbiota and Polycystic Ovary Syndrome (PCOS): A Review. Geburtshilfe Frauenheilkd..

[4] Goodman N.F., Cobin R.H., Futterweit W., Glueck J.S., Legro R., Carmina E. (2015). American Association of Clinical Endocrinologists, American College of Endocrinology, and Androgen Excess and PCOS Society Disease State Clinical Review: Guide to the Best Practices in the Evaluation and Treatment of Polycystic Ovary Syndrome—Part 1. Endocr. Pract..

[5] Khashchenko E., Vysokikh M., Uvarova E., Krechetova L., Vtorushina V., Ivanets T., Volodina M., Tarasova N., Sukhanova I., Sukhikh G. (2020). Activation of Systemic Inflammation and Oxidative Stress in Adolescent Girls with Polycystic Ovary Syndrome in Combination with Metabolic Disorders and Excessive Body Weight. J. Clin. Med..

[6] Wendland N., Opydo-Szymaczek J., Mizgier M., Jarząbek-Bielecka G. (2021). Subgingival microflora in adolescent females with polycystic ovary syndrome and its association with oral hygiene, gingivitis, and selected metabolic and hormonal parameters. Clin. Oral Investig..

[7] Wendland N., Opydo-Szymaczek J., Formanowicz D., Blacha A., Jarząbek-Bielecka G., Mizgier M. (2021). Association between metabolic and hormonal profile, proinflammatory cytokines in saliva and gingival health in adolescent females with polycystic ovary syndrome. BMC Oral Health.

[8] Deans R. (2019). Polycystic Ovary Syndrome in Adolescence. Med. Sci..

[9] Rosenfield R.L., Ehrmann D.A. (2016). The Pathogenesis of Polycystic Ovary Syndrome (PCOS): The Hypothesis of PCOS as Functional Ovarian Hyperandrogenism Revisited. Endocr. Rev..

[10] Rosenfield R.L. (2015). The Diagnosis of Polycystic Ovary Syndrome in Adolescents. Pediatrics.

[11] Kumari S., Pankaj S., Kavita K., Choudhary V., Raghwendra K.H. (2015). Study of Adolescent Girls with Irregularities for Polycystic Ovaries and Insulin Resistance. J. Evol. Med. Dent. Sci..

[12] Dumesic D.A., Oberfield S.E., Stener-Victorin E., Marshall J.C., Laven J.S., Legro R.S. (2015). Scientific Statement on the Diagnostic Criteria, Epidemiology, Pathophysiology, and Molecular Genetics of Polycystic Ovary Syndrome. Endocr. Rev..

[13] Ciebiera M., Esfandyari S., Siblini H., Prince L., Elkafas H., Wojtyła C., Al-Hendy A., Ali M. (2021). Nutrition in Gynecological Diseases: Current Perspectives. Nutrients.

[14] Greenwood E.A., Kao C.-N., Cedars M.I., Huddleston H.G. (2017). On your feet: Is sitting time linked to adverse metabolic profiles in polycystic ovary syndrome, independent of exercise?. Fertil. Steril..

[15] Moran L.J., Tassone E.C., Boyle J., Brennan L., Harrison C.L., Hirschberg A.L., Lim S., Marsh K., Misso M.L., Redman L. (2020). Evidence summaries and recommendations from the international evidence-based guideline for the assessment and management of polycystic ovary syndrome: Lifestyle management. Obes. Rev..

[16] Teede H.J., Misso M.L., Costello M.F., Dokras A., Laven J., Moran L., Piltonen T., Norman R.J. (2018). International PCOS Network. Recommendations from the international evidence-based guideline for the assessment and management of polycystic ovary syndrome. Hum. Reprod..

[17] Stamets K., Taylor D.S., Kunselman A., Demers L.M., Pelkman C.L., Legro R.S. (2004). A randomized trial of the effects of two types of short-term hypocaloric diets on weight loss in women with polycystic ovary syndrome. Fertil. Steril..

[18] Altieri P., Cavazza C., Pasqui F., Morselli A.M., Gambineri A., Pasquali R. (2012). Dietary habits and their relationship with hormones and metabolism in overweight and obese women with polycystic ovary syndrome. Clin. Endocrinol..

[19] Teede H.J., Joham A.E., Paul E., Moran L., Loxton D., Jolley D., Lombard C. (2013). Longitudinal weight gain in women identified With polycystic ovary syndrome: Results of an observational study in young women. Obesity.

[20] Eleftheriadou M., Michala L., Stefanidis K., Iliadis I., Lykeridou A., Antsaklis A. (2012). Exercise and Sedentary Habits Among Adolescents with PCOS. J. Pediatr. Adolesc. Gynecol..

[21] Mizgier M., Jarząbek-Bielecka G., Opydo-Szymaczek J., Wendland N., Więckowska B., Kędzia W. (2020). Risk Factors of Overweight and Obesity Related to Diet and Disordered Eating Attitudes in Adolescent Girls with Clinical Features of Polycystic Ovary Syndrome. J. Clin. Med..

[22] Mizgier M., Jarząbek-Bielecka G., Wendland N., Jodłowska-Siewert E., Nowicki M., Brożek A., Kędzia W., Formanowicz D., Opydo-Szymaczek J. (2021). Relation between Inflammation, Oxidative Stress, and Macronutrient Intakes in Normal and Excessive Body Weight Adolescent Girls with Clinical Features of Polycystic Ovary Syndrome. Nutrients.

[23] Stepto N.K., Patten R.K., Tassone E.C., Misso M.L., Brennan L., Boyle J., Boyle R.A., Harrison C.L., Hirschberg A.L., Marsh K. (2019). Exercise Recommendations for Women with Polycystic Ovary Syndrome: Is the Evidence Enough?. Sports Med..

[24] Kazemi M., Hadi A., Pierson R.A., Lujan M.E., Zello G.A., Chilibeck P.D. (2021). Effects of Dietary Glycemic Index and Glycemic Load on Cardiometabolic and Reproductive Profiles in Women with Polycystic Ovary Syndrome: A Systematic Review and Meta-analysis of Randomized Controlled Trials. Adv. Nutr..

[25] Azadi-Yazdi M., Karimi-Zarchi M., Salehi-Abargouei A., Fallahzadeh H., Nadjarzadeh A. (2017). Effects of Dietary Approach to Stop Hypertension diet on androgens, antioxidant status and body composition in overweight and obese women with polycystic ovary syndrome: A randomised controlled trial. J.Hum. Nutr.Diet..

[26] Toscani M.K., Mario F.M., Bagatini S.R., Wiltgen D., Matos M.C., Spritzer P.M. (2011). Effect of high-protein or normal-protein diet on weight loss, body composition, hormone, and metabolic profile in southern Brazilian women with polycystic ovary syndrome: A randomized study. Gynecol. Endocrinol..

[27] Asemi Z., Samimi M., Tabassi Z., Shakeri H., Sabihi S.-S., Esmaillzadeh A. (2014). Effects of DASH diet on lipid profiles and biomarkers of oxidative stress in overweight and obese women with polycystic ovary syndrome: A randomized clinical trial. Nutrition.

[28] Johnston B.C., Kanters S., Bandayrel K., Wu P., Naji F., Siemieniuk R.A., Ball G.D.C., Busse J., Thorlund K., Guyatt G. (2014). Comparison of Weight Loss Among Named Diet Programs in Overweight and Obese Adults. JAMA.

[29] Gardner C.D., Trepanowski J.F., Del Gobbo L.C., Hauser M.E., Rigdon J., Ioannidis J.P., Desai M., King A.C. (2018). Effect of Low-Fat vs Low-Carbohydrate Diet on 12-Month Weight Loss in Overweight Adults and the Association With Genotype Pattern or Insulin Secretion. JAMA.

[30] Papavasiliou K., Papakonstantinou E. (2017). Nutritional support and dietary interventions for women with polycystic ovary syndrome. Nutr. Diet. Suppl..

[31] Asemi Z., Esmaillzadeh A. (2014). DASH Diet, Insulin Resistance, and Serum hs-CRP in Polycystic Ovary Syndrome: A Randomized Controlled Clinical Trial. Horm. Metab. Res..

[32] Lin A.W., Kazemi M., Jarrett B.Y., Brink H.V., Hoeger K.M., Spandorfer S.D., Lujan M.E. (2019). Dietary and Physical Activity Behaviors in Women with Polycystic Ovary Syndrome per the New International Evidence-Based Guideline. Nutrients.

[33] Wong J.M.W., Gallagher M., Gooding H., Feldman H.A., Gordon C.M., Ludwig D., Ebbeling C.B. (2016). A randomized pilot study of dietary treatments for polycystic ovary syndrome in adolescents. Pediatr. Obes..

[34] Rotterdam ESHRE/ASRM-Sponsored PCOS Consensus Workshop Group (2004). Revised 2003 consensus on diagnostic criteria and long-term health risks related to polycystic ovary syndrome. Fertil Steril.

[35] Kowalkowska J., Slowinska M.A., Slowinski D., Dlugosz A., Niedzwiedzka E., Wadolowska L. (2013). Comparison of a Full Food-Frequency Questionnaire with the Three-Day Unweighted Food Records in Young Polish Adult Women: Implications for Dietary Assessment. Nutrients.

[36] Jarosz M., Rychlik E., Stoś K., Wierzejska R., Wojtasik A., Charzewska J., Mojska H., Szponar L., Sajór I., Kłosiewicz-Latoszek L. (2017). Normy Zywienia dla Populacji Polski.

[37] Gayoso-Diz P., Otero-González A., Rodriguez-Alvarez M.X., Gude F., García F., De Francisco A., Quintela A.G. (2013). Insulin resistance (HOMA-IR) cut-off values and the metabolic syndrome in a general adult population: Effect of gender and age: EPIRCE cross-sectional study. BMC Endocr. Disord..

[38] Kowalkowska J., Wadolowska L., Czarnocinska J., Czlapka-Matyasik M., Galinski G., Jezewska-Zychowicz M., Bronkowska M., Dlugosz A., Loboda D., Wyka J. (2018). Reproducibility of a Questionnaire for Dietary Habits, Lifestyle and Nutrition Knowledge Assessment (KomPAN) in Polish Adolescents and Adults. Nutrients.

[39] Faghfoori Z., Fazelian S., Shadnoush M., Goodarzi R. (2017). Nutritional management in women with polycystic ovary syndrome: A review study. Diabetes Metab. Syndr. Clin. Res. Rev..

[40] Parker D.R., Weiss S.T., Troisi R., Cassano P.A., Vokonas P.S., Landsberg L. (1993). Relationship of dietary saturated fatty acids and body habitus to serum insulin concentrations: The Normative Aging Study. Am. J. Clin. Nutr..

[41] Stender S., Dyerberg J. (2004). Influence of Trans Fatty Acids on Health. Ann. Nutr. Metab..

[42] Luo J., Rizkalla S.W., Boillot J., Alamowitch C., Chaib H., Bruzzo F., Desplanque N., Dalix A.M., Durand G., Slama G. (1996). Dietary (n-3) polyunsaturated fatty acids improve adipocyte insulin action and glucose metabolism in insu-lin-resistant rats: Relation to membrane fatty acids. J. Nutr..

[43] Sierra P., Ling P.R., Istfan N.W., Bistrian B.R. (1995). Fish oil feeding improves muscle glucose uptake in tumor necrosis factor-treated rats. Metabolism.

[44] Storlien L.H., Higgins J.A., Thomas T.C., Brown M.A., Wang H.Q., Huang X.-F., Else P. (2000). Diet composition and insulin action in animal models. Br. J. Nutr..

[45] Zivkovic A.M., German J.B., Sanyal A.J. (2007). Comparative review of diets for the metabolic syndrome: Implications for nonalcoholic fatty liver disease. Am. J. Clin. Nutr..

[46] Kasim-Karakas S.E., Almario R.U., Gregory L., Wong R., Todd H., Lasley B.L. (2004). Metabolic and Endocrine Effects of a Polyunsaturated Fatty Acid-Rich Diet in Polycystic Ovary Syndrome. J. Clin. Endocrinol. Metab..

[47] Madigan C., Ryan M., Owens D., Collins P., Tomkin G.H. (2000). Dietary unsaturated fatty acids in type 2 diabetes: Higher levels of postprandial lipoprotein on a linoleic acid-rich sunflower oil diet compared with an oleic acid-rich olive oil diet. Diabetes Care.

[48] Mizgier M., Jarzabek-Bielecka G., Mruczyk K. (2021). Maternal diet and gestational diabetes mellitus development. J. Matern. Neonatal Med..

[49] Mizgier M., Jarząbek-Bielecka G. (2014). Diabetes and sexual dysfunctions during menopause and andropause. Arch. Perinat. Med..

[50] Ryan M., McInerney D., Owens D., Collins P., Johnson A., Tomkin G. (2000). Diabetes and the Mediterranean diet: A beneficial effect of oleic acid on insulin sensitivity, adipocyte glucose transport and endothelium-dependent vasoreactivity. QJM: Int. J. Med..

[51] Gołąbek K.D., Regulska-Ilow B. (2019). Dietary support in insulin resistance: An overview of current scientific reports. Adv. Clin. Exp. Med..

[52] Mirabelli M., Chiefari E., Arcidiacono B., Corigliano D.M., Brunetti F.S., Maggisano V., Russo D., Foti D.P., Brunetti A. (2020). Mediterranean Diet Nutrients to Turn the Tide against Insulin Resistance and Related Diseases. Nutrients.

[53] Ginter E., Simko V. (2012). Plant polyphenols in prevention of heart disease. Bratisl Lek List..

[54] Mirabelli M., Russo D., Brunetti A. (2020). The Role of Diet on Insulin Sensitivity. Nutrients.

[55] Björck I., Elmståhl H.L. (2003). The glycaemic index: Importance of dietary fibre and other food properties. Proc. Nutr. Soc..

[56] U.S. Department of Health and Human Services and U.S. Department of Agriculture (2015). 2015–2020 Dietary Guidelines for Americans.

[57] Kazemi M., Pierson R.A., Lujan M.E., Chilibeck P.D., McBreairty L.E., Gordon J.J., Serrao S.B., Zello G.A., Chizen D.R. (2019). Comprehensive Evaluation of Type 2 Diabetes and Cardiovascular Disease Risk Profiles in Reproductive-Age Women with Polycystic Ovary Syndrome: A Large Canadian Cohort. J. Obstet. Gynaecol. Can..

[58] Gaskins A.J., Mumford S.L., Zhang C., Wactawski-Wende J., Hovey K.M., Whitcomb B.W., Howards P.P., Perkins N.J., Yeung E., BioCycle Study Group (2009). Effect of daily fiber intake on reproductive function: The BioCycle Study. Am. J. Clin. Nutr..

[59] Eken M.K., Ersoy G.S., Abide C.Y., Sanverdi I., Devranoglu B., Kutlu T., Cevik O. (2019). Association between circulating neuregulin 4 levels and metabolic, aterogenic, and AMH profile of polycystic ovary syndrome. J. Obstet. Gynaecol..

[60] Khayyatzadeh S.S., Kazemi-Bajestani S.M.R., Bagherniya M., Mehramiz M., Tayefi M., Ebrahimi M., Ferns G.A., Safarian M., Ghayour-Mobarhan M. (2017). Serum high C reactive protein concentrations are related to the intake of dietary macronutrients and fiber: Findings from a large representative Persian population sample. Clin. Biochem..

[61] Buyken A.E., Goletzke J., Joslowski G., Felbick A., Cheng G., Herder C., Brand-Miller J.C. (2014). Association between carbohydrate quality and inflammatory markers: Systematic review of observational and interventional studies. Am. J. Clin. Nutr..

[62] Szczuko M., Zapalowska-Chwyć M., Drozd R. (2019). A Low Glycemic Index Decreases Inflammation by Increasing the Concentration of Uric Acid and the Activity of Glutathione Peroxidase (GPx3) in Patients with Polycystic Ovary Syndrome (PCOS). Mol..

[63] Barrea L., Marzullo P., Muscogiuri G., Di Somma C., Scacchi M., Orio F., Aimaretti G., Colao A., Savastano S. (2018). Source and amount of carbohydrate in the diet and inflammation in women with polycystic ovary syndrome. Nutr. Res. Rev..

[64] Marzouk T.M., Ahmed W.A.S. (2015). Effect of Dietary Weight Loss on Menstrual Regularity in Obese Young Adult Women with Polycystic Ovary Syndrome. J. Pediatr. Adolesc. Gynecol..

[65] Moran L.J., Noakes M., Clifton P.M., Tomlinson L., Norman R. (2003). Dietary Composition in Restoring Reproductive and Metabolic Physiology in Overweight Women with Polycystic Ovary Syndrome. J. Clin. Endocrinol. Metab..

[66] Moran L.J., Noakes M., Clifton P., Wittert G.A., Williams G., Norman R. (2006). Short-term meal replacements followed by dietary macronutrient restriction enhance weight loss in polycystic ovary syndrome. Am. J. Clin. Nutr..

[67] Soares N.P., Dos Santos A.C.S., Costa E.C., Azevedo G.D., Damasceno D.C., Fayh A.P.T., Lemos T. (2016). Diet-Induced Weight Loss Reduces DNA Damage and Cardiometabolic Risk Factors in Overweight/Obese Women with Polycystic Ovary Syndrome. Ann. Nutr. Metab..

[68] Marsh K.A., Steinbeck K.S., Atkinson F., Petocz P., Brand-Miller J. (2010). Effect of a low glycemic index compared with a conventional healthy diet on polycystic ovary syndrome. Am. J. Clin. Nutr..

[69] Mehrabani H.H., Salehpour S., Amiri Z., JalaliFarahani S., Meyer B.J., Tahbaz F. (2012). Beneficial Effects of a High-Protein, Low-Glycemic-Load Hypocaloric Diet in Overweight and Obese Women with Polycystic Ovary Syndrome: A Randomized Controlled Intervention Study. J. Am. Coll. Nutr..

[70] Barr S., Reeves S., Sharp K., Jeanes Y. (2013). An Isocaloric Low Glycemic Index Diet Improves Insulin Sensitivity in Women with Polycystic Ovary Syndrome. J. Acad. Nutr. Diet..

[71] Harrison C.L., Lombard C., Moran L., Teede H.J. (2010). Exercise therapy in polycystic ovary syndrome: A systematic review. Hum. Reprod. Update.

[72] Almenning I., Rieber-Mohn A., Lundgren K.M., Løvvik T.S., Garnæs K.K., Moholdt T. (2015). Effects of High Intensity Interval Training and Strength Training on Metabolic, Cardiovascular and Hormonal Outcomes in Women with Polycystic Ovary Syndrome: A Pilot Study. PLoS ONE.

[73] Khashchenko E.P., Sukhanova S.A., Pyataeva S.V., Volodina M.A., Tarasova N.V., Tsvirkun D.V., Uvarova E.V., Vysokikh M.Y. (2017). Indicators of mitochondrial functioning in adolescent girls with polycystic ovary syndrome with regard to the presence of metabolic disorders and overweight. Akusherstvo Ginekol.

[74] Thompson F.E., Kirkpatrick S.I., Subar A.F., Reedy J., Schap T.E., Wilson M.M., Krebs-Smith S.M. (2015). The National Cancer Institute’s Dietary Assessment Primer: A Resource for Diet Research. J. Acad. Nutr. Diet..

[75] McCarney R., Warner J., Iliffe S., Van Haselen R., Griffin M., Fisher P. (2007). The Hawthorne Effect: A randomised, controlled trial. BMC Med. Res. Methodol..

[76] Kanaya N., Vonderfecht S., Chen S. (2013). Androgen (dihydrotestosterone)-mediated regulation of food intake and obesity in female mice. J. Steroid Biochem. Mol. Biol..

